# Usefulness of semi-automatic harmonization strategy of standardized uptake values for multicenter PET studies

**DOI:** 10.1038/s41598-021-87942-0

**Published:** 2021-04-19

**Authors:** Hiromitsu Daisaki, Kazuhiro Kitajima, Masatoyo Nakajo, Tadashi Watabe, Kimiteru Ito, Fumi Sakamoto, Tadaki Nakahara, Mana Ishibashi, Akira Toriihara

**Affiliations:** 1 Graduate School of Radiological Technology, Gunma Prefectural College of Health Science, 323-1 Kamioki-machi, Maebashi, Gunma 371-0052 Japan; 2 Graduate School of Medical and Dental Sciences, Tokyo Medical and Dental University, 1-5-45 Yushima, Bunkyo-ku, Tokyo, 113-8510 Japan; 3 Division of Nuclear Medicine and PET Center, Department of Radiology, Hyogo College of Medicine, 1-1 Mukogawa-cho, Nishinomiya, Hyogo 663-8501 Japan; 4 Department of Radiology, Graduate School of Medical and Dental Sciences, Kagoshima University, 8-35-1, Sakuragaoka, Kagoshima, 890-8544 Japan; 5 Department of Nuclear Medicine and Tracer Kinetics, Graduate School of Medicine, Osaka University, 2-2 Yamadaoka, Suita, Osaka 565-0871 Japan; 6 Department of Diagnostic Radiology, National Cancer Center Hospital, 5-1-1 Tsukiji, Chuo-ku, Tokyo, 104-0045 Japan; 7 Department of Diagnostic Radiology, Graduate School of Life Sciences, Kumamoto University, 1-1-1 Honjo, Chuo-ku, Kumamoto, 860-8556 Japan; 8 Department of Diagnostic Radiology, Keio University School of Medicine, 35 Shinanomachi, Shinjuku-ku, Tokyo, 160-8582 Japan; 9 Division of Radiology, Department of Pathophysiological and Therapeutic Sciences, Tottori University, 86 Nishi-cho, Yonago, Tottori, 683-8503 Japan; 10 PET Imaging Center, Asahi General Hospital, 1326 I, Asahi, Chiba 289-2511 Japan

**Keywords:** Oncology, Cancer, Cancer imaging

## Abstract

This study assessed the possibility of semi-automatic harmonization of standardized uptake values (SUVs) in multicenter studies. Phantom data were acquired using 16 PET/CT scanners (including 3 PET/CT scanners with a silicon photomultiplier detector). PET images obtained using 30-min/bed scans for optimum harmonization filter calculations and using 90–180-s/bed scans for SUV validation under clinical conditions were obtained. Time of flight and a reconstruction method with point-spread function correction were allowed. The optimal full width at half maximum of the 3D-Gaussian filter that minimizes the root mean square error with the median value of the JSNM harmonization range was calculated semi-automatically. The SUVmax and the SUVpeak of the hot spheres were measured, and the inter-scanner coefficient of variation (COV) was calculated before and after harmonization. The harmonization filter was applied to 11 of the 15 PET/CT scanners in which the SUV calibration accuracy had been verified, but not in the remaining 4 scanners. Under noiseless conditions before harmonization, the inter-scanner COVs of the SUVmax and the SUVpeak were as high as 21.57% and 12.20%, respectively, decreasing to 8.79% and 5.73% after harmonization, respectively. Harmonization brought the SUVmax of all the hot spheres to within the harmonization range. Even under clinical conditions affected by image noise, the inter-scanner COVs for the SUVmax and SUVpeak were as high as 8.83% and 5.18% after harmonization, respectively. By applying an optimal harmonization filter that is calculated semi-automatically, the harmonization of SUVs according to the JSNM strategy is possible in multicenter studies, thereby reducing inter-scanner COVs.

## Introduction

Positron emission tomography (PET)/computed tomography (CT) using 2-deoxy-2-(18F)fluoro-D-glucose (FDG) is useful for the initial diagnosis, staging, restaging, monitoring, and prediction of prognosis of various types of tumors^1–3^. Quantitative PET evaluations are generally performed using the standardized uptake value (SUV). By defining two-dimensional (2D) regions of interest (ROIs) or three-dimensional (3D) volumes of interest (VOIs) on PET images, various types of SUVs can be calculated automatically as follows: the SUVmax, which is the maximum value in the ROI; the SUVpeak, which is the average value in a local area of 1 cm^3^ surrounding the voxel with the maximum tracer uptake; and the SUVmean, which is the average SUV calculated from voxels that exceed a particular SUV threshold. The SUVmax is the most frequently used parameter in clinical settings because of a good inter-observer agreement and robustness against a partial volume effect (PVE). On the other hand, the SUVmax is susceptible to an upward bias arising from image noise, which can easily fluctuate depending on the PET scanners and protocols that are used. Lodge et al. reported that the SUVpeak is more robust to image noise than the SUVmax in PET images^4^. However, the SUVpeak can exhibit a large variation because of the ambiguous definition of its measuring method, and this may influence the assessment of responses to therapy^5^. Recently, the Quantitative Imaging Biomarker Alliance (QIBA) defined a method for calculating SUVpeak^6^. For the SUVpeak measure, it is necessary to automatically and reproducibly detect the place where the mean value in the 1 cm^3^ region is the highest within the placed VOI. Therefore, a strategy for standardizing methods of calculating the SUV is necessary to validate PET/CT as a quantitative tool and the SUV as an imaging biomarker^7^.

To facilitate multicenter studies using FDG-PET, EANM Research Ltd. (EARL) previously reported the upper and lower limits of the recovery coefficient and proposed the concept of “harmonization of SUVs”^8^. Considering recent technological innovations, such as new reconstruction technologies (i.e., point spread function [PSF]) and the practical application of semiconductor PET/CT using silicon photomultiplier (SiPM) detectors, an additional filter, known as a harmonization filter, could be applied for SUV harmonization^9^. The use of a software tool (EQ.PET) that harmonizes SUVs among different PET systems has highlighted the possibility of SUV harmonization using a harmonization filter, suggesting the possibility of both good tumor detectability and quantitative harmonization^10^. Generally, a 3D-Gaussian filter is used as a harmonization filter for software with a harmonizing function, and the parameters provided by the full width at half maximum (FWHM) must be changed to adjust the SUV. Lasnon et al. reported that SUV harmonization according to the EARL strategy is possible in multicenter studies by optimizing the FWHM of the 3D-Gaussian filter based on the root mean square error (RMSE) for the target SUV using phantom data^11^.

In the J-Hart study conducted in Japan, Tsutsui et al. set the SUVmax calculated by applying a 3D-Gaussian filter of 10 mm at FWHM to a Digital Reference Object (DRO) created by QIBA as the target of harmonization; in this manner, they showed that the harmonization of quantitative values is possible using phantom data acquired in a multicenter study^12^. The Japanese Society of Nuclear Medicine (JSNM) has established a phantom test method in which the upper and lower limits of the SUVmax are described for the purpose of harmonizing quantitative values in multicenter studies^13^. Daisaki et al. reported a multicenter study of malignant lymphoma that adopted a standardization process for image quality in accordance with the JSNM guidelines before the harmonization strategy was proposed^14^. Since then, no reports have been found in Japan regarding multicenter studies focusing on quantitative evaluations involving the application of JSNM's harmonization strategy.

We attempted to harmonize SUVs according to JSNM's strategy using a semi-automatic harmonization method in a multicenter study. We also examined the reproducibility of quantitative indicators (SUVmax and SUVpeak) in PET images under clinical conditions affected by image noise.

## Materials and methods

### Ethics

The phantom data used in this study were obtained for a retrospective multicenter study of the assessing treatment effects and the prediction of treatment effect of immune checkpoint inhibitors. The clinical PET images presented in this paper are from a study approved by ethics committee of the Hyogo College of Medicine (No. 3315), which waived the requirement for informed consent. This study was conducted in accordance with the Declaration of Helsinki and Ethical Guidelines for Medical and Health Research Involving Human Subjects.

### Phantom data for harmonization

PET images were acquired from 16 PET/CT scanners in 8 institutions, including 3 PET/CT scanners equipped with SiPM detectors. According to the JSNM guidelines, a radioactivity of ^18^F solution (Hot:BG ratio of 4:1 for all PET/CT scanners) determined according to the injection dose of each institution was enclosed in the image quality phantom^15^ (Table 1). To check the cross-calibration accuracy, the average SUV in the phantom BG region was measured using 12 ROIs with a diameter of 37 mm and was verified to be within the range of 1.00 ± 0.05. One PET/CT scanner was excluded from this study because the average SUV showed a low value beyond the acceptable range.

**Table 1:** Image reconstruction parameters under clinical conditions. The application of the latest technologies, such as PSF reconstruction and TOF, were allowed.

	Biograph Duo	Biograph 64 mCT TrueV	Biograph 40 mCT TrueV	Discovery 600 (No.1)	Discovery 600 (No.2)	Discovery MI 4R (No.1)	Discovery MI 4R (No.2)	Discovery MI DR
Background Activity (Bq/mL)	2.51	2.65	2.65	2.65	2.65	2.65	2.65	2.53
Reconstruction Method	FORE-OSEM	3D-OSEM	3D-OSEM	3D-OSEM	3D-OSEM	Q.Clear	Q.Clear	Q.Clear
Matrix Size	128 × 128	256 × 256	256 × 256	192 × 192	192 × 192	192 × 192	256 × 256	192 × 192
Voxel size (mm)	5.30 × 5.30 × 3.38	3.18 × 3.18 × 2.03	3.18 × 3.18 × 2.03	3.13 × 3.13 × 3.27	2.08 × 2.08 × 3.27	3.13 × 3.13 × 2.79	2.73 × 2.73 × 2.79	3.13 × 3.13 × 3.27
Iteration	2	3	3	2	2	−	−	−
Subset	8	21	21	16	16	−	−	−
penalization factor (β)	−	−	−	−	−	700	700	650
Smoothing filter	Gaussian	Gaussian	Gaussian	Gaussian	Gaussian	−	−	−
Z-axis filter (for GE scanners)	−	−	−	Standard	Standard	N/A	N/A	N/A
FWHM of filter (mm)	5	6	6	4	5	−	−	−
PSF correction	−	On	On	−	−	On	On	On
TOF	−	On	On	−	−	On	On	On
Acquisition duration in clinical condition (sec/bed)	120	120	120	150	120	150	120	90 – 180

	Discovery 710	Discovery IQ 5R	Aquiduo	GEMINI GX-L (No.1)	GEMINI TF	Ingenuity TF	Vereos	
Background Activity (Bq/mL)	2.65	2.66	2.65	2.57	2.63	2.84	2.69	
Reconstruction Method	3D-OSEM	Q.Clear	FORE-OSEM	LOR-RAMLA	3D-OSEM	3D-OSEM	3D-OSEM	
Matrix Size	192 × 192	192 × 192	128 × 128	144 × 144	144 × 144	144 × 144	144 × 144	
Voxel size (mm)	3.65 × 3.65 × 3.27	3.13 × 3.13 × 3.26	3.98 × 3.98 × 2.00	4.00 × 4.00 × 4.00	4.00 × 4.00 × 4.00	4.00 × 4.00 × 4.00	4.00 × 4.00 × 4.00	
Iteration	3	−	4	2	3	3	3	
Subset	8	−	14	−	33	33	15	
penalization factor (β)	−	400	−	−	−	−	−	
Smoothing filter	Gaussian	−	Gaussian	Relaxation Parameter = 0.5	Relaxation Parameter = 0.5	Relaxation Parameter = 1.0	−	
Z-axis filter (for GE scanners)	Standard	N/A	−	−	−	−	−	
FWHM of filter (mm)	4	−	8	−	−	−	−	
PSF correction	On	On	−	−	−	−	−	
TOF	On	−	−	−	On	On	On	
Acquisition duration in clinical condition (sec/bed)	120	180	120	90	90	90	90	

All the PET emission data were acquired using a 30 min/bed scan in 3D list-mode. CT data were also acquired using each institution’s default parameters and were used for attenuation correction of the PET images. The list-mode data for the 30 min/bed scans were reconstructed using each institution’s default parameters for optimal harmonization filter calculations. Table 1 shows the image reconstruction method and parameters for each scanner. In this study, PSF correction for superior lesion detectability was allowed throughout the image reconstruction process.

### Semi-automatic SUV harmonization method

**Figure 1:**
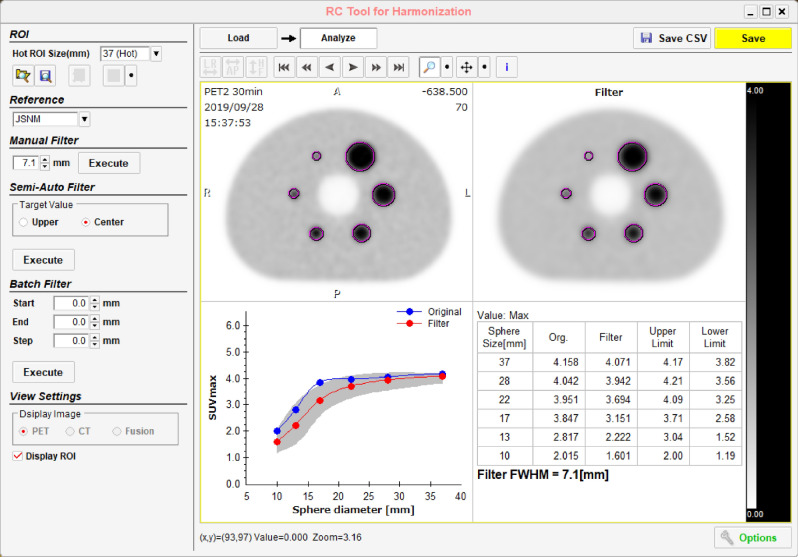
RMSE-based additional filter optimization using the RC Tool for Harmonization.

As shown in Fig. 1, the RC Tool for Harmonization (Nihon Medi-Physics Co., Ltd.) was used to set the ROI at the same size as the diameters of all the hot spheres and to calculate the SUVmax. The optimum FWHM of the harmonization filter was defined as the value that minimizes the RMSE calculated using Eq. (1).

1 $$RMSE = \sqrt {\frac{1}{6}\mathop \sum \limits_{i = 10,13,17,22,28,37} \left( {targetSUV_{max,i} - filterSUV_{max,i} } \right)^{2} }$$

**Figure 2:**
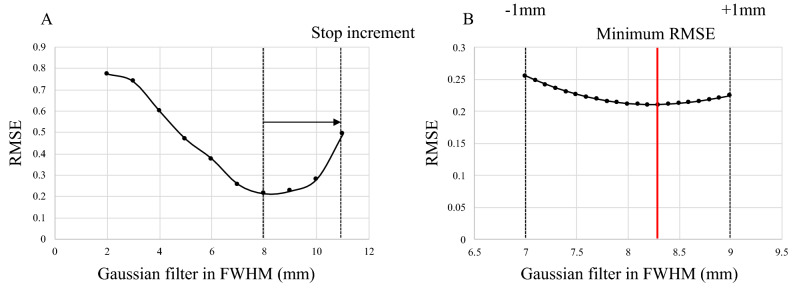
Optimization strategy used by the RC Tool for Harmonization.

Here, targetSUVmax was the median value of the harmonization range defined in the JSNM guidelines. The filterSUVmax was measured using a PET image with a 3D-Gaussian filter applied to the PET image with the default parameters of each institution, and the 3D-Gaussian filter was incremented by 1 mm from 2 to 10 mm at FWHM. In addition, when the RMSE for the last 3 mm recorded an increase, the FWHM increment to be applied was terminated. Subsequently, the RMSE was calculated in 0.1 mm increments within the range of ± 1 mm of the FWHM value that achieved a minimum RMSE value in 1-mm increments (Fig. 2). The FWHM of the optimum harmonization filter with one decimal place was automatically displayed in the RC Tool for Harmonization. When the RMSE increased by applying the 3D-Gaussian filter, compared with the RMSE calculated using each institution’s default parameter, N/A was displayed in the RC Tool for Harmonization, and the PET image without the harmonization filter was judged to be optimal.

### Coefficient of variation (COV) among scanners

**Figure 3:**
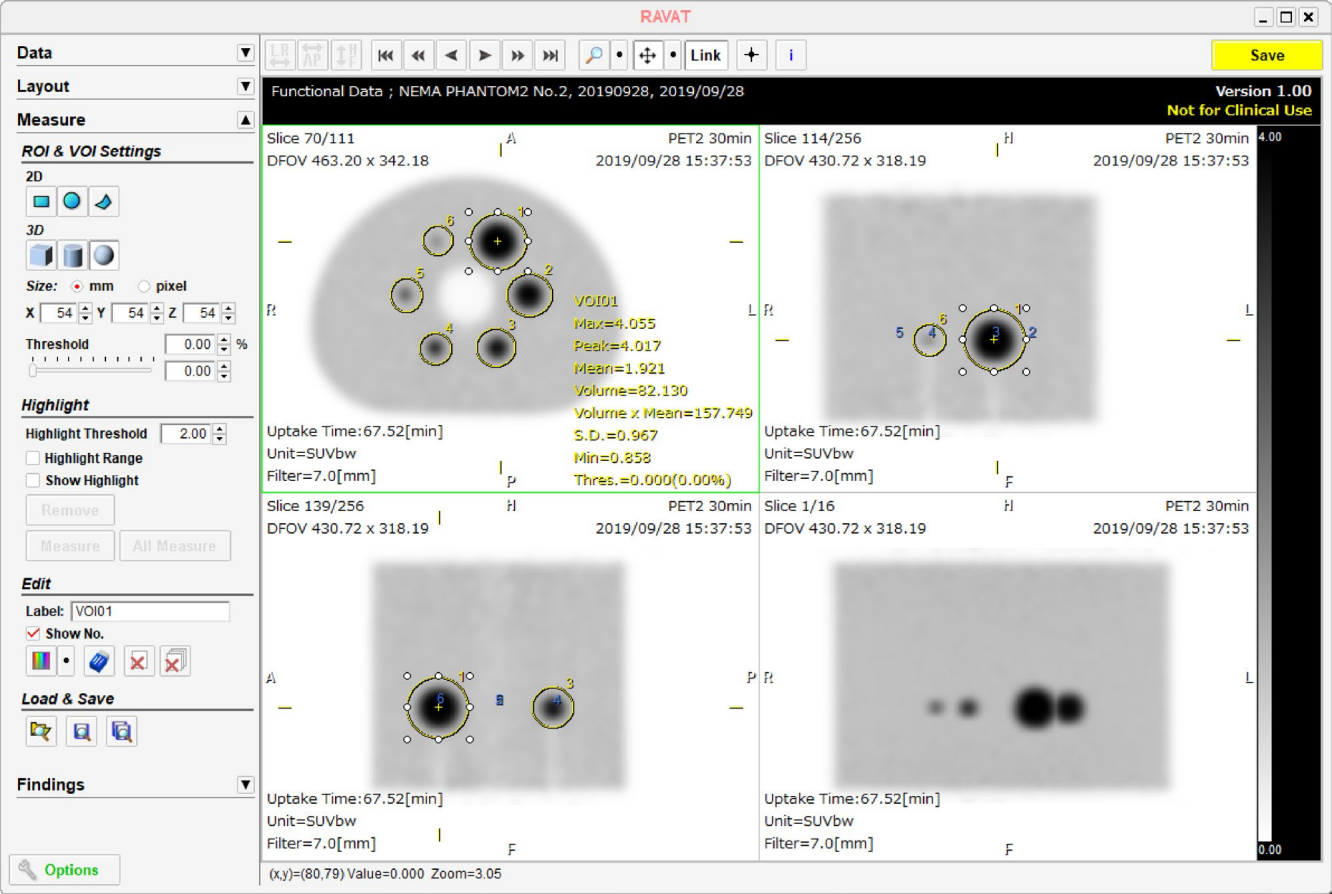
VOI placement for SUV validation using RAVAT.

The list-mode PET emission data was re-binned according to the acquisition duration used clinically, and image reconstruction was performed using each institution’s default reconstruction parameters. The SUVmax and SUVpeak were calculated before and after harmonization using both PET images from the 30 min/bed scan, which is less affected by image noise, and those obtained using the clinical acquisition duration typically used at each institution. The SUVs were calculated by setting a VOI of the same size as each hot sphere using RAVAT (Nihon Medi-Physics Co., Ltd.), which is a PET quantitative analysis software for research that is compliant with the QIBA profile (Fig. 3). The SUVpeak of the hot sphere was measured except for 10 mm (taking into account the measurement definition). The differences between the scanners before and after harmonization were evaluated using the COV shown in Eq. (2).

2 $$COV = \frac{SD}{{mean}} \times 100 \left( \% \right)$$

## Results

### Changes in RMSE according to changes in FWHM produced by additional 3D-Gaussian filters

**Figure 4:**
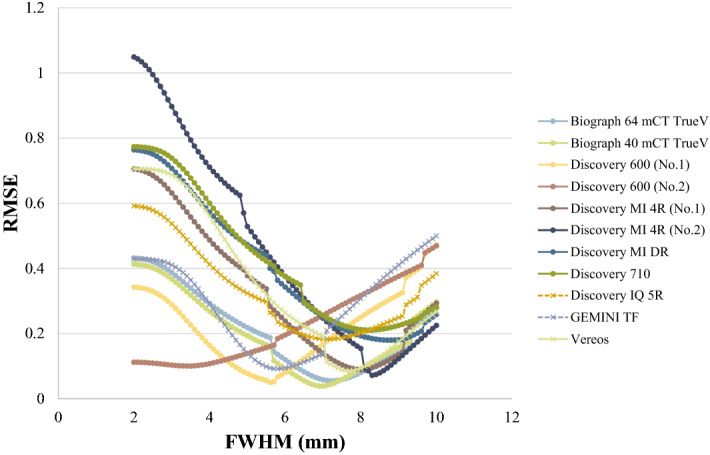
Changes in RMSE produced by changes in FWHM of 3D-Gaussian filters.

Figure 4 shows the RMSE when the 3D-Gaussian filter was changed in increments from 2 to 10 mm. By applying the optimization strategy of the 3D-Gaussian filter using the RC Tool for Harmonization, the optimum FWHM of the harmonization filter producing the lowest RMSE value was determined for 11 of the 15 scanners in which the SUV calibration accuracy had been verified. The remaining 4 scanners had the lowest RMSE in each institution’s default PET images in which a 3D-Gaussian filter was not applied.

### SUVmax and SUVpeak before and after harmonization

**Figure 5:**
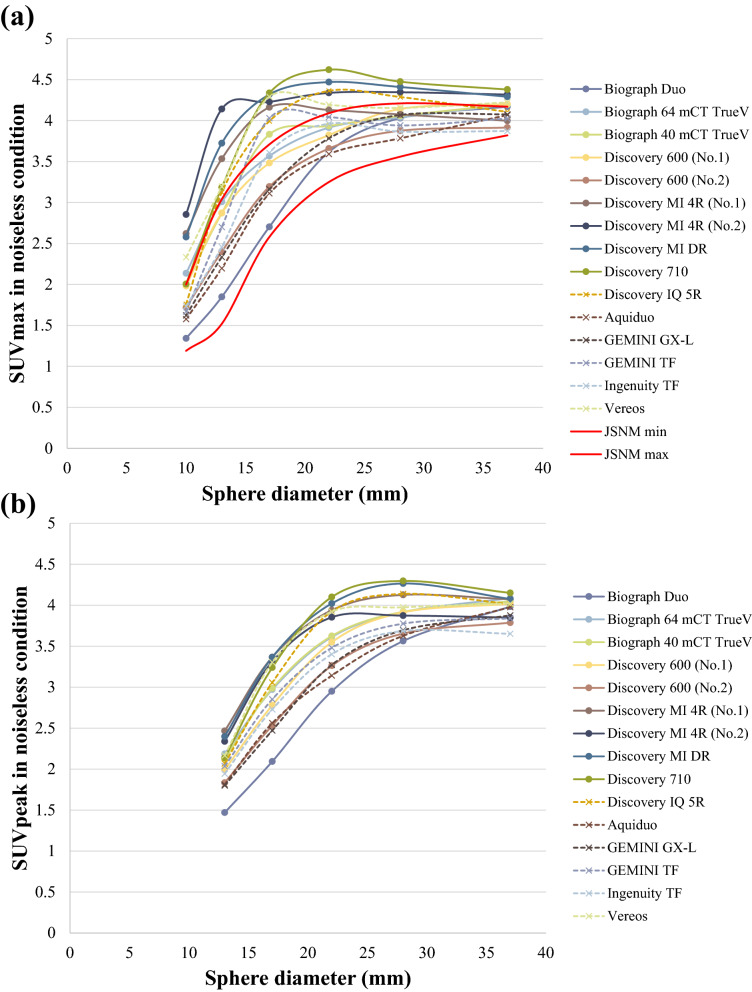
(**a**) SUVmax and (**b**) SUVpeak under each institution’s reconstruction parameters and noiseless conditions. The solid red lines show the upper and lower limits of the SUVmax defined according to the JSNM guidelines. Some scanners had an SUVmax that was higher than the upper limit. The harmonization range of the SUVpeak is not defined in the JSNM guidelines. The actual values obtained using each scanner are shown in Online Resource 1 (SUVmax) and Online Resource 2 (SUVpeak).

Figure 5a, b respectively show the SUVmax and the SUVpeak calculated for the PET images reconstructed according to each institution’s default parameters for the 30 min/bed (noiseless conditions) scans. For SUVmax, the COVs of the differences among the scanners were 21.57%, 20.49%, 14.4%, 8.24%, 4.71%, and 3.13% for the 10-, 13-, 17-, 22-, 28-, and 37-mm hot spheres, respectively. For the SUVpeak, the COVs were 12.20%, 12.71%, 9.41%, 5.76%, and 3.29% for the 13-, 17-, 22-, 28-, and 37-mm hot spheres, respectively.

**Figure 6:**
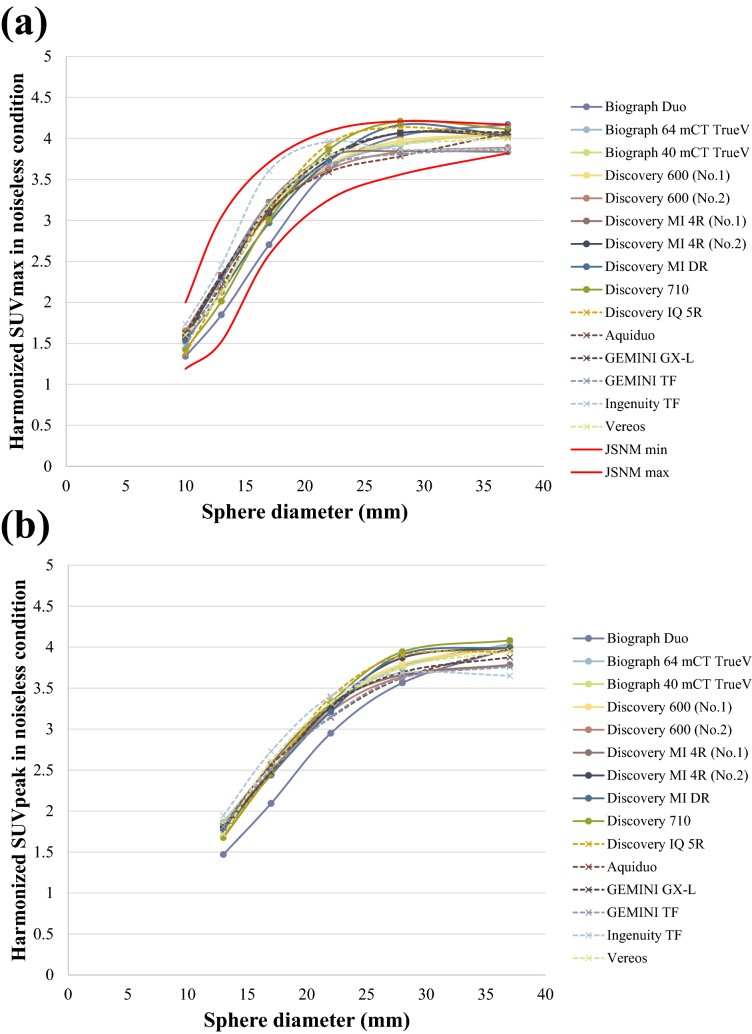
(**a**) Harmonized SUVmax and (**b**) harmonized SUVpeak after the addition of an optimal harmonization filter under noiseless conditions. The solid red lines show the upper and lower limits of the SUVmax defined according to the JSNM guidelines. The harmonized SUVmax were within the harmonization range of the JSNM guidelines for all the scanners. The actual values obtained using each scanner are shown in Online Resource 3 (harmonized SUVmax) and Online Resource 4 (harmonized SUVpeak).

Figure 6a, b respectively show the harmonized SUVmax and harmonized SUVpeak calculated for the PET images obtained during the 30 min/bed scans after the application of the optimal 3D-Gaussian filter derived from the RC Tool for Harmonization. The SUVmax for all the hot spheres of all the scanners fell within the JSNM’s harmonization range. For the harmonized SUVmax, the COVs of the differences among the scanners were 8.79%, 8.54%, 6.34%, 4.60%, 6.63%, and 6.37% for the 10-, 13-, 17-, 22-, 28-, and 37-mm hot spheres, respectively. For the harmonized SUVpeak, the COVs were 5.73%, 5.23%, 3.31%, 3.04%, and 3.03% for the 13-, 17-, 22-, 28-, and 37-mm hot spheres, respectively.

**Figure 7:**
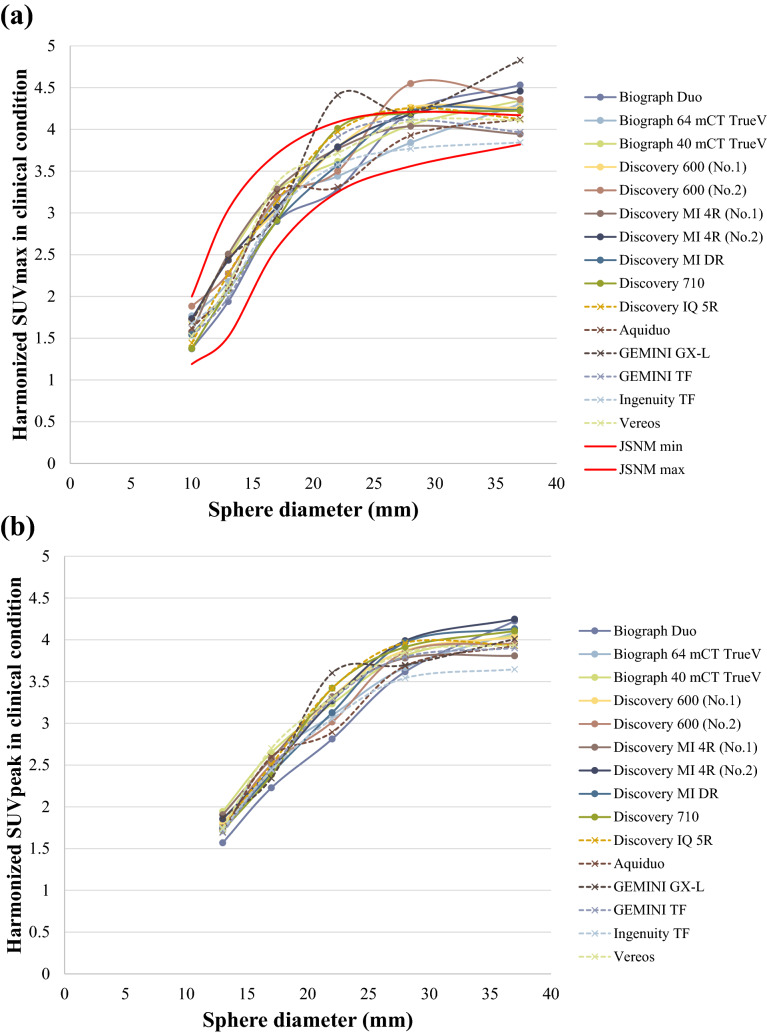
(**a**) Harmonized SUVmax and (**b**) harmonized SUVpeak after the addition of an optimal harmonization filter under clinical conditions. The harmonized SUVmax measured using PET images under clinical conditions exceeded the JSNM upper limits for the 22-mm, 28-mm, and 37-mm hot spheres on some scanners. The actual values obtained using each scanner are shown in Online Resource 5 (harmonized SUVmax) and Online Resource 6 (harmonized SUVpeak).

Figure 7a, b respectively show the harmonized SUVmax and harmonized SUVpeak calculated for PET images obtained using the acquisition duration typically used for clinical studies at each institution. For the harmonized SUVmax, the COVs of the differences among the scanners were 8.83%, 8.32%, 4.68%, 7.65%, 4.45%, and 5.68% for the 10-, 13-, 17-, 22-, 28-, and 37-mm hot spheres, respectively. For the harmonized SUVpeak, the COVs were 5.18%, 4.85%, 6.35%, 3.43%, and 3.75% for the 13-, 17-, 22-, 28-, and 37-mm hot spheres, respectively.

The actual SUVmax, SUVpeak, and optimal harmonization filters calculated for each PET/CT scanner are shown in the online resources (1–6).

### Clinical images with or without additional harmonization filter

**Figure 8:**
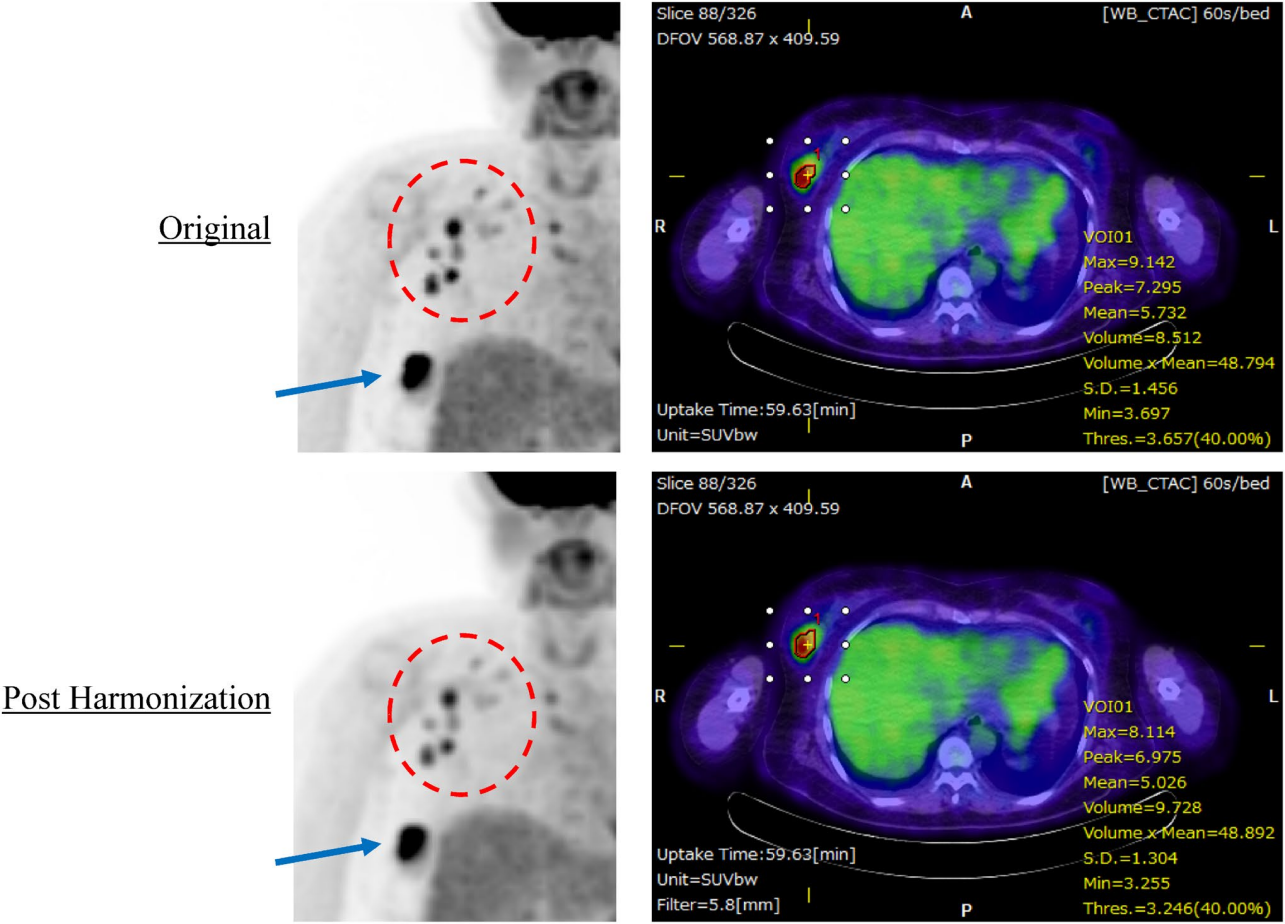
A case with accumulation of FDG in the right primary breast cancer and metastatic lymph nodes. Maximum intensity projection images (left column) and fused PET/CT images (right column) before and after harmonization are shown. The fused images show quantitative values calculated by applying a threshold of 40% to VOI using RAVAT for right primary breast cancer lesions.

The PET images before and after applying the harmonization filter with FWHM = 5.8 mm were shown in Fig. 8. The quantitative values of the primary breast cancer were SUVmax = 9.14, SUVpeak = 7.30 before the harmonization, and SUVmax = 8.11, SUVpeak = 6.98 after the harmonization. Since the resolution of PET images is usually reduced by the harmonization, the edges of the lesions are slightly blurred (the area within the red dotted line), but the detection rate of lesions is not significantly affected in this patient.

## Discussion

In this study, we investigated whether harmonization strategy defined by JSNM could be applied to heterogenous multicenter studies involving PET/CT scanners of different generations including SiPM PET/CT scanners. First, to perform harmonization with a high reproducibility and objectivity, the optimum FWHM of the 3D-Gaussian filter for harmonization was calculated semi-automatically using the RC Tool for Harmonization targeting the median value of the harmonization range specified by the JSNM^13^. Next, if necessary, the optimal harmonization filter was applied to PET images having different degrees of image noise that had been reconstructed using each institution’s default reconstruction parameters.

The SUVmax measured using each institution’s default reconstruction parameters were not included in the harmonization range of the JSNM strategy for 10 of the 15 PET/CT scanners. This multicenter study included both conventional PET/CT scanners with PMT detectors and semiconductor PET/CT scanners with SiPM detectors. Some of the included PET/CT scanners used an image reconstruction method with PSF correction, which helps to improve lesion detectability. However, PSF correction can also result in an overestimation of the SUV because of edge artifacts (or Gibbs artifacts)^16^. Thus, the JSNM’s current harmonization strategy is defined based on PET image data from multicenter studies without PSF correction. PET images obtained using the image reconstruction parameters typically used clinically at each institution are often ineligible for quantitative evaluations in multicenter studies. In the present study, we determined that all the SUVmax that were originally judged to be ineligible according to the JSNM harmonization strategy could be included in the defined range by applying the optimal harmonization filter that was semi-automatically calculated using the research software RC Tool for Harmonization. Furthermore, harmonization decreased the maximum COV of the SUVmax among the PET/CT scanners from 21.57 to 8.79% at a 10-mm hot sphere. Harmonization also decreased the maximum COV of the SUVpeak from 12.71% at a 17-mm hot sphere to 5.73% at a 13-mm hot sphere. Tsutsui et al. reported the J-Hart study, which attempted to harmonize 12 PET/CT scanners using the target SUVmax calculated by a DRO, and the FWHM of the optimum harmonization filter was calculated for each scanner by changing the 3D-Gaussian filter by 1 mm^12^. In their study, a maximum COV of 10.7% was observed for a 13-mm sphere even after harmonization. In the present study, however, the COV was less than 10% despite the more heterogeneous multicenter study design and the inclusion of semiconductor PET/CT scanners. This reduction in COV may be due to the fact that the RC Tool for Harmonization semi-automatically optimizes the harmonization filter in units of 0.1 mm. Furthermore, in the present study, the SUVpeak was also measured based on the QIBA’s measurement principle and a better COV of 5.73%, compared with the SUVmax, was achieved.

In this study, we also estimated the practical differences among PET/CT scanners under clinical conditions by applying a calculated optimum harmonization filter to PET images obtained using the clinical acquisition duration typically used at each institution. Although the harmonized SUVmax showed that the results for hot spheres of 22–37 mm in diameter fell outside the harmonization range because of the influence of an upward bias caused by image noise, the COV was within 10% (highest value was 8.83% for a 10-mm hot sphere), which was comparable to the COVs (highest value of 8.79% for a 10-mm hot sphere) of harmonized SUVmax based on PET images obtained under noiseless conditions. The maximum COV of the harmonized SUVpeak calculated from PET images obtained under clinical conditions was 6.35%, which was lower than the COV of the harmonized SUVmax. In other words, quantitative multicenter studies with acceptable inter-scanner variability could be feasible if software-based harmonization method and SUVpeak quantification are applied.

The reproducibility of analyses and analysis software is important in quantitative studies involving the analysis of PET images. In the past, ambiguities in calculation processes have been problematic when a SUVpeak was recommended, instead of the noise-sensitive SUVmax^5^. Specifically, whether the pixel for the SUVmax calculation should be the center of the SUVpeak measurement or whether the SUVpeak should be the output after searching for the region where the SUVpeak was highest in all places within the tumor is unclear. In the harmonization process as well, a clear objective definition and a high reproducibility are required when applying a harmonization filter. The RC Tool for Harmonization used in this study uses 1-mm increments in the range of 0–20 mm to identify the minimum RMSE value and then calculates the RMSE in 0.1-mm increments within the minimum value ± 1 mm range. In the present study, detailed harmonization filter optimization was possible by finding the minimum RMSE value. Of note, this process is highly reproducible and has the clarity of the derivation process of RMSE-based optimization, as there are no manual interventions by the analyst.

Although not found in this study, if the SUVmax calculated in the PET image with before harmonization falls below the lower limit of the harmonization range, it is necessary to reconsider the image reconstruction condition (generally, iteration, subset and filter). Furthermore, if there is an event, such as changing acquisition and image reconstruction parameters, upgrading software, reducing the sensitivity of PET/CT scanner and the accompanying overhaul, etc., that affects the image quality or quantitative accuracy of the original PET image, it is necessary to re-verify whether SUVmax is within the harmonization range. Thus, the constancy of PET images needs to be checked at regular basis. The presence of a centralized analysis laboratory in a multicenter PET study should be expected to yield better results for SUV harmonization and also helps to provide quality assurance that is important for clinical research.

JSNM defines physical image quality standards (e.g., CV < 10%) for determining imaging conditions in the JSNM guidelines, mainly to improve the quality of clinical research and clinical trials. The phantom data of this study was acquired based on the clinical routine protocol of each institution, except for the calculation of the optimal harmonization filter. As a result, even though the model were the same (e.g., Discovery MI 4R No. 1 and No. 2 in this study), one could be harmonized appropriately, while another could not. Moreover, as shown in Fig. 7a, the curve of SUVmax can fluctuate up and down unstably even after the harmonization. Since this phenomenon was not observed in the PET image with sufficient acquisition duration as shown in Fig. 5a, the fluctuation of the curve might have caused by a statistical noise. In the harmonization method using post filtering, the quantitative value may fluctuate depending on the quality of the original PET image to be processed. Therefore, it is desirable that the original PET image meets a certain image quality standard such as CV < 10%. It is necessary to verify the accuracy of SUV harmonization using the proposed harmonization method by applying it to phantom data that meets the image quality standards established by JSNM.

Research on the standardization and harmonization of quantitative values for PET has been led and promoted by the Society of Nuclear Medicine/Clinical Trial Network^17,18^ and EANM/EARL^19,20^ for a long time prior to JSNM. Kaalep have reported a feasibility study on SUV harmonization in PET/CT scanner with advanced TOF and PSF technologies^21^, and the harmonization range of EARL's accreditation program has been updated in advance of JSNM guideline^22^. In the future, the harmonization filter will no longer be necessary for PET/CT scanners with these advanced technologies by the update of JSNM's harmonization range. On the other hand, PET/CT scanners without PSF or TOF technology may be excluded from quantitative multicenter studies due to their inability to adapt to the harmonization range that will be updated. In other words, it should be noted that research on SUV harmonization for multicenter PET studies will be updated as appropriate in the future.

Of the 16 PET/CT scanners registered in this study, one PET/CT scanner was excluded because the SUV calibration accuracy was not verified. Since the PET/CT scanner data was obtained retrospectively, the cause of the error that occurred when the phantom was acquired could not be clarified in this study. In addition to the possibility of a cross-calibration error, there is also the possibility of an error in the phantom data acquisition procedure. The accuracy of routine QC/QA processes and the accurate implementation of phantom data acquisition are important for the accurate achievement of harmonization in multicenter studies.

## Conclusions

Quantitative harmonized multicenter studies according to the JSNM strategy are achievable by applying optimization strategy of a harmonization filter calculated semi-automatically, even in heterogeneous multicenter studies involving different generations of PET/CT scanners. When conducting harmonized multicenter studies involving quantitative evaluations of PET images, differences among scanners can be further reduced by using the SUVpeak instead of the SUVmax.

## Supplementary Information

Supplementary Information.

Supplementary Information Legends.

## Data Availability

All the analyses were performed using RC Tool for Harmonization (Nihon Medi-Physics Co.,Ltd.) and RAVAT (Nihon Medi-Physics Co.,Ltd.). Although membership registration is required, download is available at following Japanese website: https://www.nmp.co.jp/member/hiroba/index.html. Phantom data are not publicly available for download, but might be retrieved from the principal investigator Hiromitsu Daisaki.
