# Supplementary material for: Usefulness of semi-automatic harmonization strategy of standardized uptake values for multicenter PET studies
Source: Sci Rep. 2021 Apr 19;11:8517. doi: 10.1038/s41598-021-87942-0 (PMC8055685; doi:10.1038/s41598-021-87942-0)
Supplement: Supplementary file 1 — Supplementary Information. [file 41598_2021_87942_MOESM1_ESM.pptx]

## Slide 1
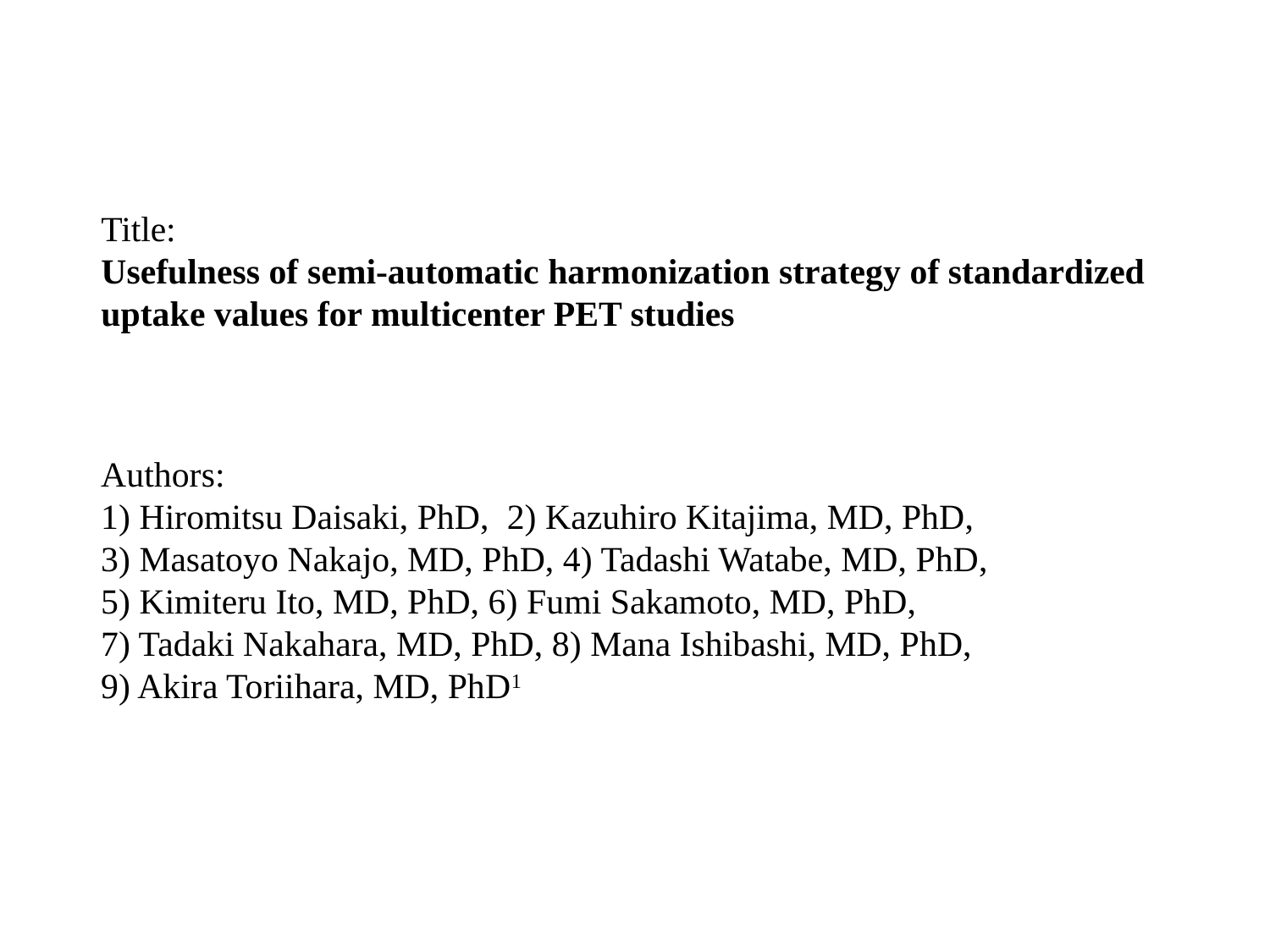

Title:
Usefulness of semi-automatic harmonization strategy of standardized uptake values for multicenter PET studies
Authors:
1) Hiromitsu Daisaki, PhD, 2) Kazuhiro Kitajima, MD, PhD, 3) Masatoyo Nakajo, MD, PhD, 4) Tadashi Watabe, MD, PhD, 5) Kimiteru Ito, MD, PhD, 6) Fumi Sakamoto, MD, PhD, 7) Tadaki Nakahara, MD, PhD, 8) Mana Ishibashi, MD, PhD, 9) Akira Toriihara, MD, PhD1

## Slide 2
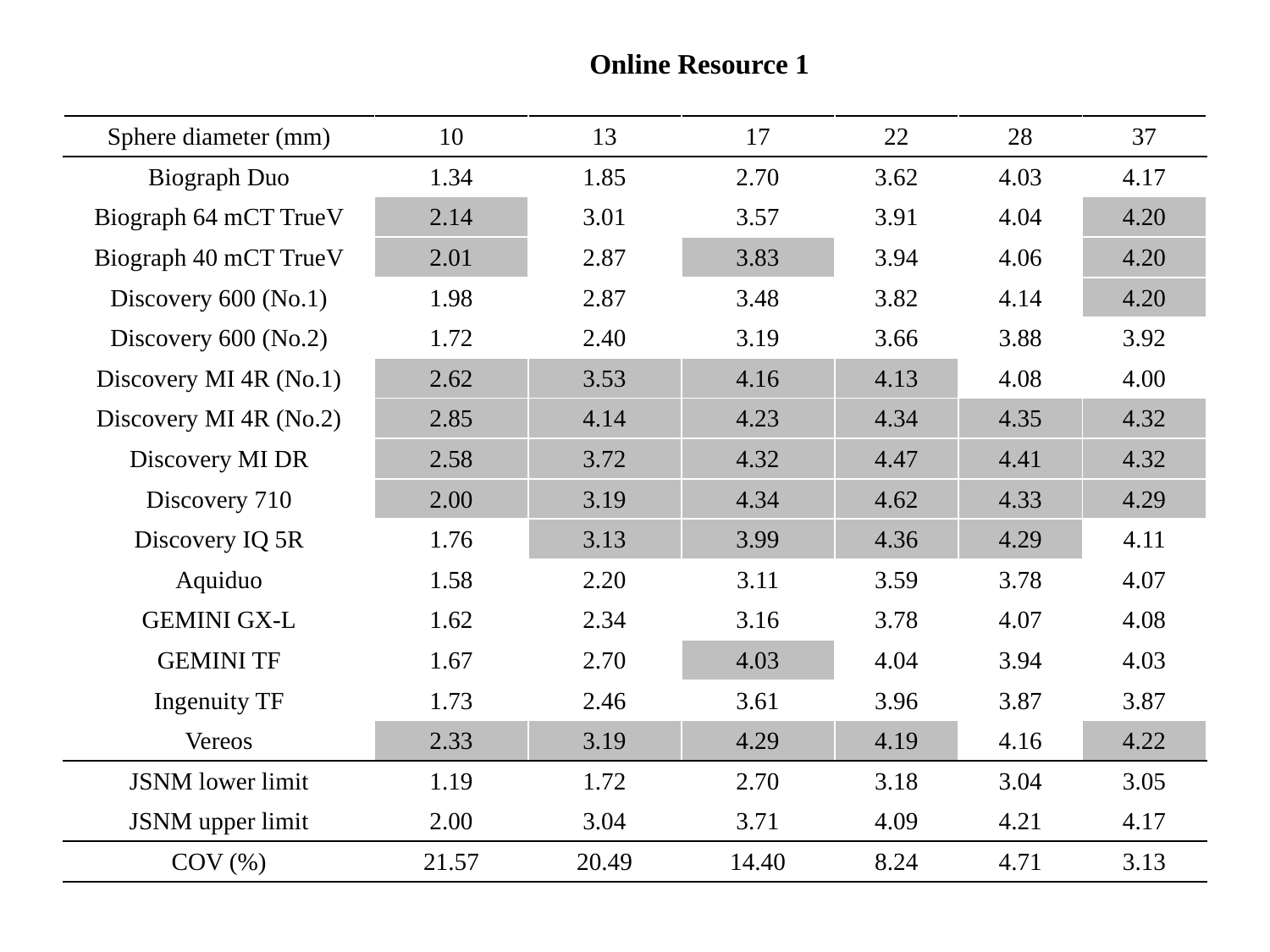

# Online Resource 1
| Sphere diameter (mm) | 10 | 13 | 17 | 22 | 28 | 37 |
| --- | --- | --- | --- | --- | --- | --- |
| Biograph Duo | 1.34 | 1.85 | 2.70 | 3.62 | 4.03 | 4.17 |
| Biograph 64 mCT TrueV | 2.14 | 3.01 | 3.57 | 3.91 | 4.04 | 4.20 |
| Biograph 40 mCT TrueV | 2.01 | 2.87 | 3.83 | 3.94 | 4.06 | 4.20 |
| Discovery 600 (No.1) | 1.98 | 2.87 | 3.48 | 3.82 | 4.14 | 4.20 |
| Discovery 600 (No.2) | 1.72 | 2.40 | 3.19 | 3.66 | 3.88 | 3.92 |
| Discovery MI 4R (No.1) | 2.62 | 3.53 | 4.16 | 4.13 | 4.08 | 4.00 |
| Discovery MI 4R (No.2) | 2.85 | 4.14 | 4.23 | 4.34 | 4.35 | 4.32 |
| Discovery MI DR | 2.58 | 3.72 | 4.32 | 4.47 | 4.41 | 4.32 |
| Discovery 710 | 2.00 | 3.19 | 4.34 | 4.62 | 4.33 | 4.29 |
| Discovery IQ 5R | 1.76 | 3.13 | 3.99 | 4.36 | 4.29 | 4.11 |
| Aquiduo | 1.58 | 2.20 | 3.11 | 3.59 | 3.78 | 4.07 |
| GEMINI GX-L | 1.62 | 2.34 | 3.16 | 3.78 | 4.07 | 4.08 |
| GEMINI TF | 1.67 | 2.70 | 4.03 | 4.04 | 3.94 | 4.03 |
| Ingenuity TF | 1.73 | 2.46 | 3.61 | 3.96 | 3.87 | 3.87 |
| Vereos | 2.33 | 3.19 | 4.29 | 4.19 | 4.16 | 4.22 |
| JSNM lower limit | 1.19 | 1.72 | 2.70 | 3.18 | 3.04 | 3.05 |
| JSNM upper limit | 2.00 | 3.04 | 3.71 | 4.09 | 4.21 | 4.17 |
| COV (%) | 21.57 | 20.49 | 14.40 | 8.24 | 4.71 | 3.13 |

## Slide 3
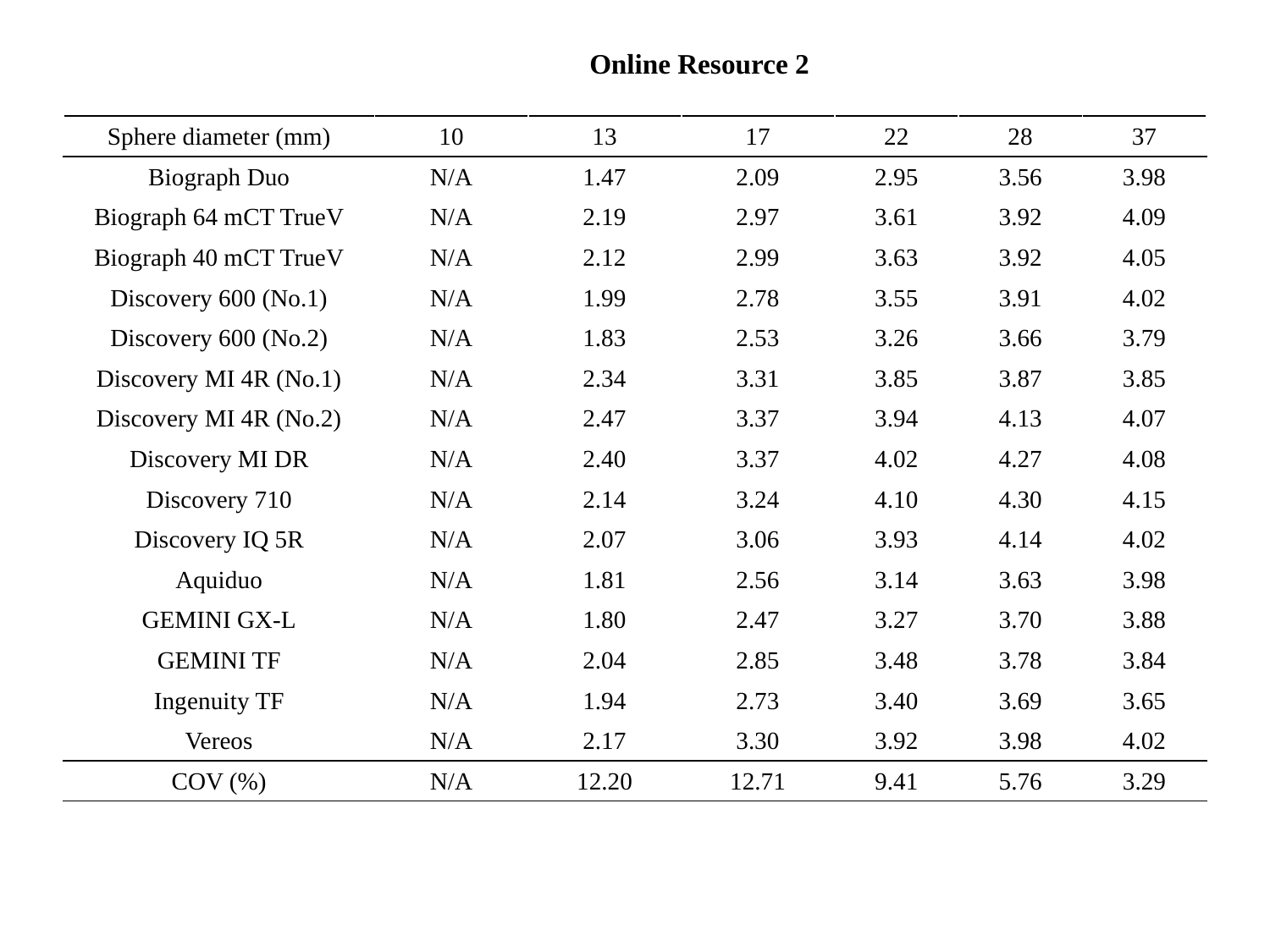

# Online Resource 2
| Sphere diameter (mm) | 10 | 13 | 17 | 22 | 28 | 37 |
| --- | --- | --- | --- | --- | --- | --- |
| Biograph Duo | N/A | 1.47 | 2.09 | 2.95 | 3.56 | 3.98 |
| Biograph 64 mCT TrueV | N/A | 2.19 | 2.97 | 3.61 | 3.92 | 4.09 |
| Biograph 40 mCT TrueV | N/A | 2.12 | 2.99 | 3.63 | 3.92 | 4.05 |
| Discovery 600 (No.1) | N/A | 1.99 | 2.78 | 3.55 | 3.91 | 4.02 |
| Discovery 600 (No.2) | N/A | 1.83 | 2.53 | 3.26 | 3.66 | 3.79 |
| Discovery MI 4R (No.1) | N/A | 2.34 | 3.31 | 3.85 | 3.87 | 3.85 |
| Discovery MI 4R (No.2) | N/A | 2.47 | 3.37 | 3.94 | 4.13 | 4.07 |
| Discovery MI DR | N/A | 2.40 | 3.37 | 4.02 | 4.27 | 4.08 |
| Discovery 710 | N/A | 2.14 | 3.24 | 4.10 | 4.30 | 4.15 |
| Discovery IQ 5R | N/A | 2.07 | 3.06 | 3.93 | 4.14 | 4.02 |
| Aquiduo | N/A | 1.81 | 2.56 | 3.14 | 3.63 | 3.98 |
| GEMINI GX-L | N/A | 1.80 | 2.47 | 3.27 | 3.70 | 3.88 |
| GEMINI TF | N/A | 2.04 | 2.85 | 3.48 | 3.78 | 3.84 |
| Ingenuity TF | N/A | 1.94 | 2.73 | 3.40 | 3.69 | 3.65 |
| Vereos | N/A | 2.17 | 3.30 | 3.92 | 3.98 | 4.02 |
| COV (%) | N/A | 12.20 | 12.71 | 9.41 | 5.76 | 3.29 |

## Slide 4
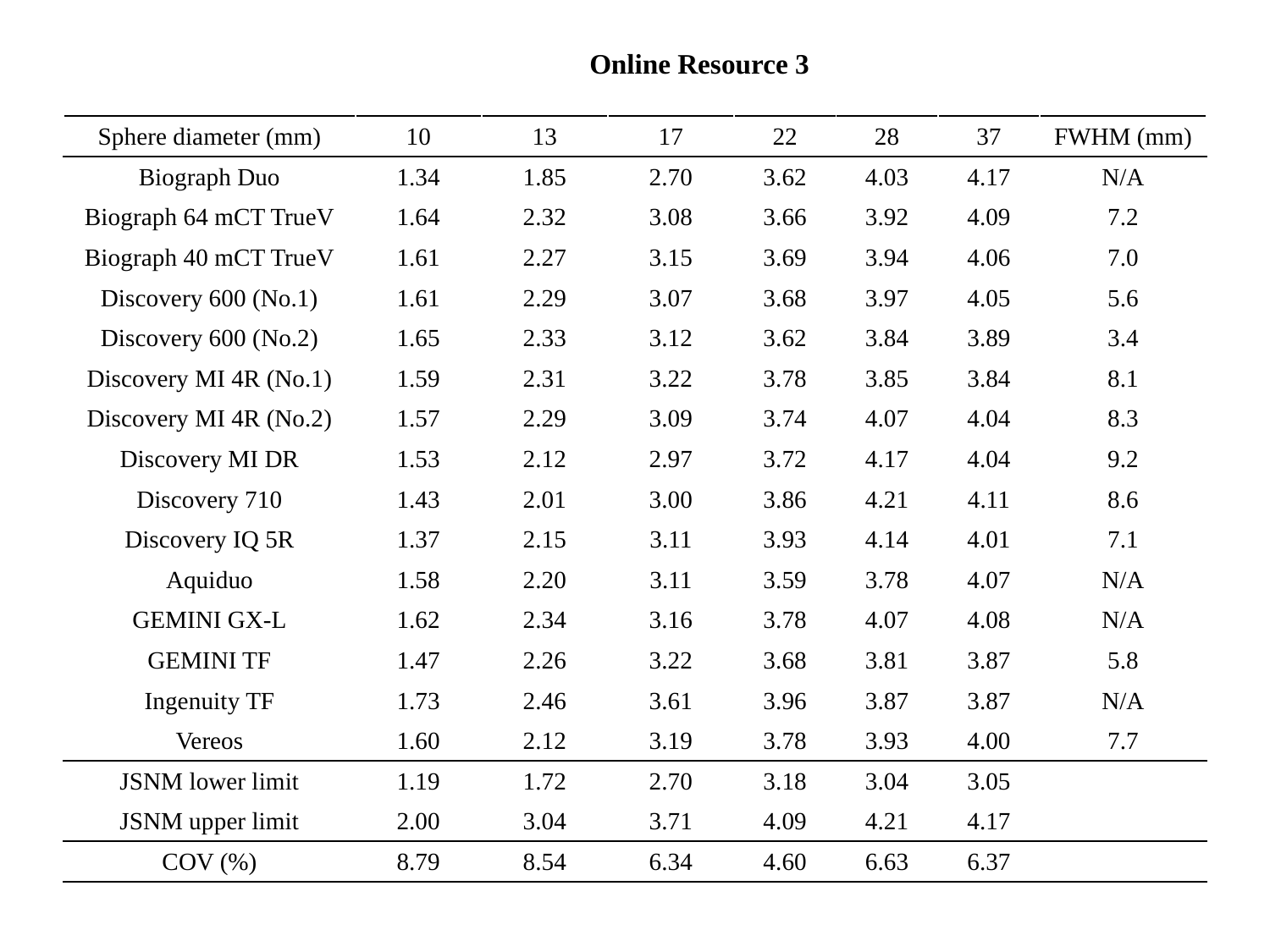

# Online Resource 3
| Sphere diameter (mm) | 10 | 13 | 17 | 22 | 28 | 37 | FWHM (mm) |
| --- | --- | --- | --- | --- | --- | --- | --- |
| Biograph Duo | 1.34 | 1.85 | 2.70 | 3.62 | 4.03 | 4.17 | N/A |
| Biograph 64 mCT TrueV | 1.64 | 2.32 | 3.08 | 3.66 | 3.92 | 4.09 | 7.2 |
| Biograph 40 mCT TrueV | 1.61 | 2.27 | 3.15 | 3.69 | 3.94 | 4.06 | 7.0 |
| Discovery 600 (No.1) | 1.61 | 2.29 | 3.07 | 3.68 | 3.97 | 4.05 | 5.6 |
| Discovery 600 (No.2) | 1.65 | 2.33 | 3.12 | 3.62 | 3.84 | 3.89 | 3.4 |
| Discovery MI 4R (No.1) | 1.59 | 2.31 | 3.22 | 3.78 | 3.85 | 3.84 | 8.1 |
| Discovery MI 4R (No.2) | 1.57 | 2.29 | 3.09 | 3.74 | 4.07 | 4.04 | 8.3 |
| Discovery MI DR | 1.53 | 2.12 | 2.97 | 3.72 | 4.17 | 4.04 | 9.2 |
| Discovery 710 | 1.43 | 2.01 | 3.00 | 3.86 | 4.21 | 4.11 | 8.6 |
| Discovery IQ 5R | 1.37 | 2.15 | 3.11 | 3.93 | 4.14 | 4.01 | 7.1 |
| Aquiduo | 1.58 | 2.20 | 3.11 | 3.59 | 3.78 | 4.07 | N/A |
| GEMINI GX-L | 1.62 | 2.34 | 3.16 | 3.78 | 4.07 | 4.08 | N/A |
| GEMINI TF | 1.47 | 2.26 | 3.22 | 3.68 | 3.81 | 3.87 | 5.8 |
| Ingenuity TF | 1.73 | 2.46 | 3.61 | 3.96 | 3.87 | 3.87 | N/A |
| Vereos | 1.60 | 2.12 | 3.19 | 3.78 | 3.93 | 4.00 | 7.7 |
| JSNM lower limit | 1.19 | 1.72 | 2.70 | 3.18 | 3.04 | 3.05 | |
| JSNM upper limit | 2.00 | 3.04 | 3.71 | 4.09 | 4.21 | 4.17 | |
| COV (%) | 8.79 | 8.54 | 6.34 | 4.60 | 6.63 | 6.37 | |

## Slide 5
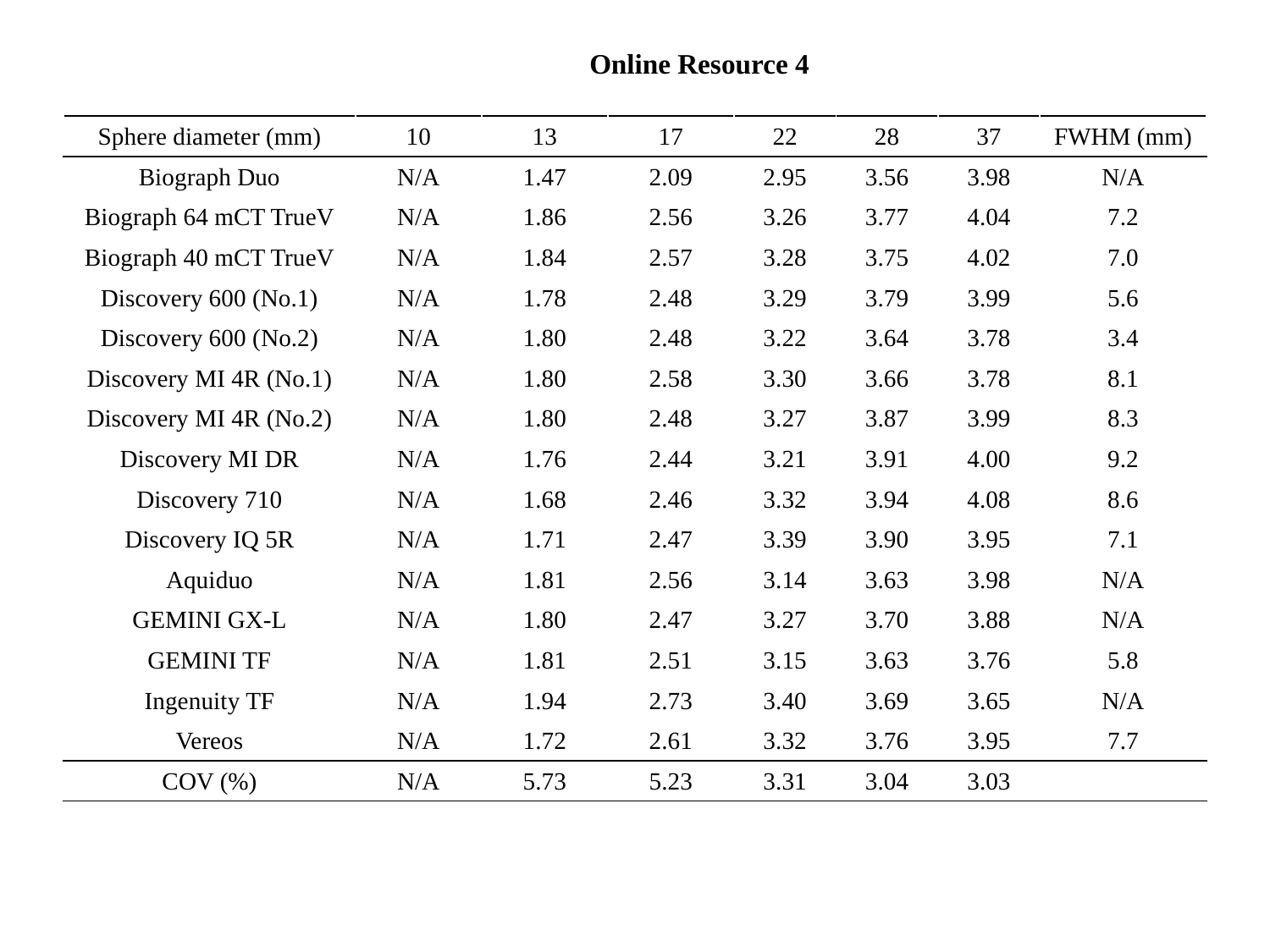

# Online Resource 4
| Sphere diameter (mm) | 10 | 13 | 17 | 22 | 28 | 37 | FWHM (mm) |
| --- | --- | --- | --- | --- | --- | --- | --- |
| Biograph Duo | N/A | 1.47 | 2.09 | 2.95 | 3.56 | 3.98 | N/A |
| Biograph 64 mCT TrueV | N/A | 1.86 | 2.56 | 3.26 | 3.77 | 4.04 | 7.2 |
| Biograph 40 mCT TrueV | N/A | 1.84 | 2.57 | 3.28 | 3.75 | 4.02 | 7.0 |
| Discovery 600 (No.1) | N/A | 1.78 | 2.48 | 3.29 | 3.79 | 3.99 | 5.6 |
| Discovery 600 (No.2) | N/A | 1.80 | 2.48 | 3.22 | 3.64 | 3.78 | 3.4 |
| Discovery MI 4R (No.1) | N/A | 1.80 | 2.58 | 3.30 | 3.66 | 3.78 | 8.1 |
| Discovery MI 4R (No.2) | N/A | 1.80 | 2.48 | 3.27 | 3.87 | 3.99 | 8.3 |
| Discovery MI DR | N/A | 1.76 | 2.44 | 3.21 | 3.91 | 4.00 | 9.2 |
| Discovery 710 | N/A | 1.68 | 2.46 | 3.32 | 3.94 | 4.08 | 8.6 |
| Discovery IQ 5R | N/A | 1.71 | 2.47 | 3.39 | 3.90 | 3.95 | 7.1 |
| Aquiduo | N/A | 1.81 | 2.56 | 3.14 | 3.63 | 3.98 | N/A |
| GEMINI GX-L | N/A | 1.80 | 2.47 | 3.27 | 3.70 | 3.88 | N/A |
| GEMINI TF | N/A | 1.81 | 2.51 | 3.15 | 3.63 | 3.76 | 5.8 |
| Ingenuity TF | N/A | 1.94 | 2.73 | 3.40 | 3.69 | 3.65 | N/A |
| Vereos | N/A | 1.72 | 2.61 | 3.32 | 3.76 | 3.95 | 7.7 |
| COV (%) | N/A | 5.73 | 5.23 | 3.31 | 3.04 | 3.03 | |

## Slide 6
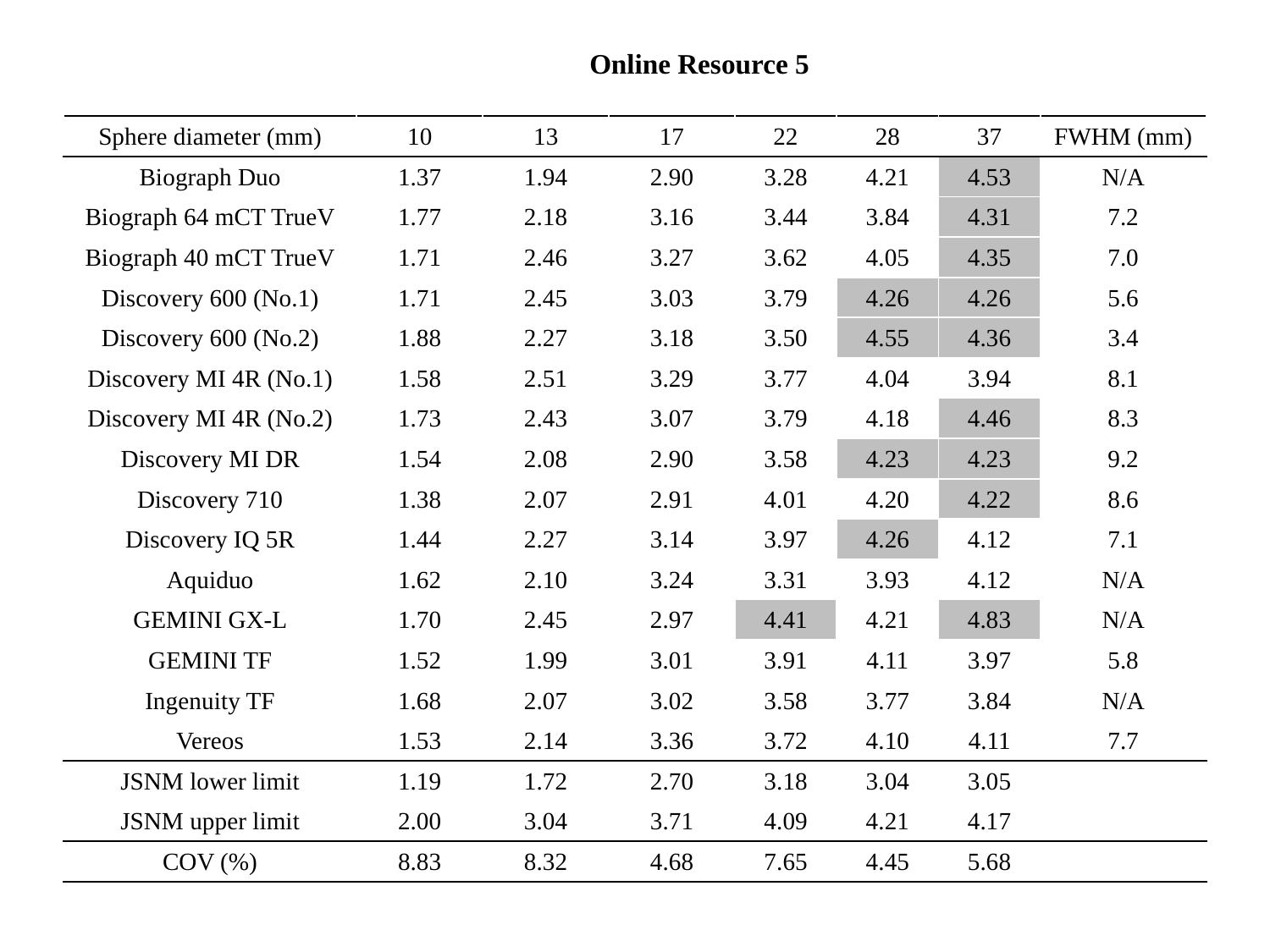

# Online Resource 5
| Sphere diameter (mm) | 10 | 13 | 17 | 22 | 28 | 37 | FWHM (mm) |
| --- | --- | --- | --- | --- | --- | --- | --- |
| Biograph Duo | 1.37 | 1.94 | 2.90 | 3.28 | 4.21 | 4.53 | N/A |
| Biograph 64 mCT TrueV | 1.77 | 2.18 | 3.16 | 3.44 | 3.84 | 4.31 | 7.2 |
| Biograph 40 mCT TrueV | 1.71 | 2.46 | 3.27 | 3.62 | 4.05 | 4.35 | 7.0 |
| Discovery 600 (No.1) | 1.71 | 2.45 | 3.03 | 3.79 | 4.26 | 4.26 | 5.6 |
| Discovery 600 (No.2) | 1.88 | 2.27 | 3.18 | 3.50 | 4.55 | 4.36 | 3.4 |
| Discovery MI 4R (No.1) | 1.58 | 2.51 | 3.29 | 3.77 | 4.04 | 3.94 | 8.1 |
| Discovery MI 4R (No.2) | 1.73 | 2.43 | 3.07 | 3.79 | 4.18 | 4.46 | 8.3 |
| Discovery MI DR | 1.54 | 2.08 | 2.90 | 3.58 | 4.23 | 4.23 | 9.2 |
| Discovery 710 | 1.38 | 2.07 | 2.91 | 4.01 | 4.20 | 4.22 | 8.6 |
| Discovery IQ 5R | 1.44 | 2.27 | 3.14 | 3.97 | 4.26 | 4.12 | 7.1 |
| Aquiduo | 1.62 | 2.10 | 3.24 | 3.31 | 3.93 | 4.12 | N/A |
| GEMINI GX-L | 1.70 | 2.45 | 2.97 | 4.41 | 4.21 | 4.83 | N/A |
| GEMINI TF | 1.52 | 1.99 | 3.01 | 3.91 | 4.11 | 3.97 | 5.8 |
| Ingenuity TF | 1.68 | 2.07 | 3.02 | 3.58 | 3.77 | 3.84 | N/A |
| Vereos | 1.53 | 2.14 | 3.36 | 3.72 | 4.10 | 4.11 | 7.7 |
| JSNM lower limit | 1.19 | 1.72 | 2.70 | 3.18 | 3.04 | 3.05 | |
| JSNM upper limit | 2.00 | 3.04 | 3.71 | 4.09 | 4.21 | 4.17 | |
| COV (%) | 8.83 | 8.32 | 4.68 | 7.65 | 4.45 | 5.68 | |

## Slide 7
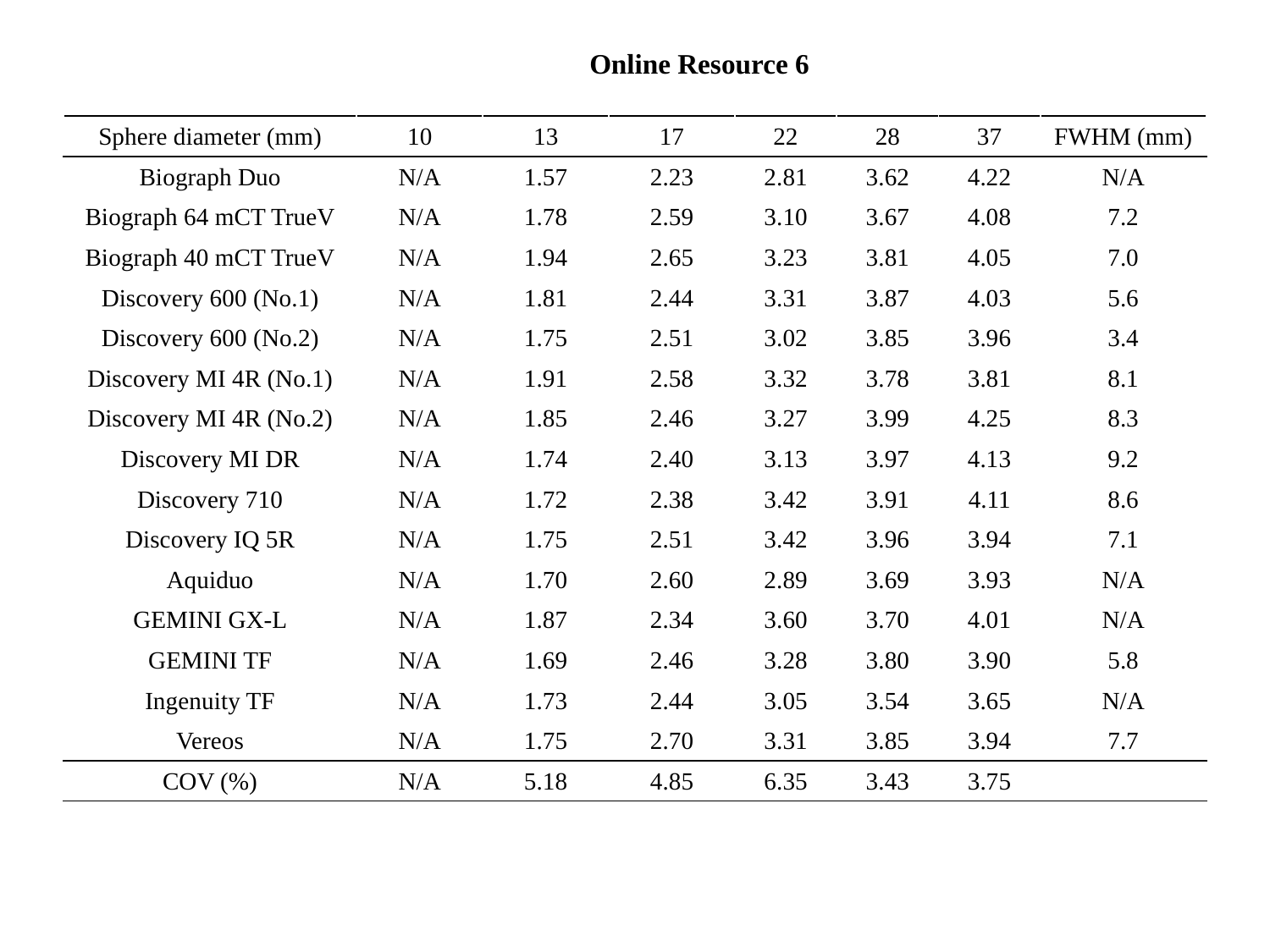

# Online Resource 6
| Sphere diameter (mm) | 10 | 13 | 17 | 22 | 28 | 37 | FWHM (mm) |
| --- | --- | --- | --- | --- | --- | --- | --- |
| Biograph Duo | N/A | 1.57 | 2.23 | 2.81 | 3.62 | 4.22 | N/A |
| Biograph 64 mCT TrueV | N/A | 1.78 | 2.59 | 3.10 | 3.67 | 4.08 | 7.2 |
| Biograph 40 mCT TrueV | N/A | 1.94 | 2.65 | 3.23 | 3.81 | 4.05 | 7.0 |
| Discovery 600 (No.1) | N/A | 1.81 | 2.44 | 3.31 | 3.87 | 4.03 | 5.6 |
| Discovery 600 (No.2) | N/A | 1.75 | 2.51 | 3.02 | 3.85 | 3.96 | 3.4 |
| Discovery MI 4R (No.1) | N/A | 1.91 | 2.58 | 3.32 | 3.78 | 3.81 | 8.1 |
| Discovery MI 4R (No.2) | N/A | 1.85 | 2.46 | 3.27 | 3.99 | 4.25 | 8.3 |
| Discovery MI DR | N/A | 1.74 | 2.40 | 3.13 | 3.97 | 4.13 | 9.2 |
| Discovery 710 | N/A | 1.72 | 2.38 | 3.42 | 3.91 | 4.11 | 8.6 |
| Discovery IQ 5R | N/A | 1.75 | 2.51 | 3.42 | 3.96 | 3.94 | 7.1 |
| Aquiduo | N/A | 1.70 | 2.60 | 2.89 | 3.69 | 3.93 | N/A |
| GEMINI GX-L | N/A | 1.87 | 2.34 | 3.60 | 3.70 | 4.01 | N/A |
| GEMINI TF | N/A | 1.69 | 2.46 | 3.28 | 3.80 | 3.90 | 5.8 |
| Ingenuity TF | N/A | 1.73 | 2.44 | 3.05 | 3.54 | 3.65 | N/A |
| Vereos | N/A | 1.75 | 2.70 | 3.31 | 3.85 | 3.94 | 7.7 |
| COV (%) | N/A | 5.18 | 4.85 | 6.35 | 3.43 | 3.75 | |
