# Supplementary material for: Usefulness of semi-automatic harmonization strategy of standardized uptake values for multicenter PET studies
Source: Sci Rep. 2021 Apr 19;11:8517. doi: 10.1038/s41598-021-87942-0 (PMC8055685; doi:10.1038/s41598-021-87942-0)
Supplement: Supplementary file 2 — Supplementary Information Legends. [file 41598_2021_87942_MOESM2_ESM.docx]

Online Resource 1.

SUVmax under each institution’s default parameters and noiseless conditions.

Online Resource 2.

SUVpeak under each institution’s default parameters and noiseless conditions.

Online Resource 3.

Harmonized SUVmax after the addition of an optimal harmonization filter under noiseless conditions.

Online Resource 4.

Harmonized SUVpeak after the addition of an optimal harmonization filter under noiseless conditions.

Online Resource 5.

Harmonized SUVmax after the addition of an optimal harmonization filter under clinical conditions.

Online Resource 6.

Harmonized SUVpeak after the addition of an optimal harmonization filter under clinical conditions.
